# A CMOS Self-Contained Quadrature Signal Generator for SoC Impedance Spectroscopy

**DOI:** 10.3390/s18051382

**Published:** 2018-04-30

**Authors:** Alejandro Márquez, Jorge Pérez-Bailón, Belén Calvo, Nicolás Medrano, Pedro A. Martínez

**Affiliations:** Group of Electronic Design, Aragon Institute for Engineering Research (GDE-I3A), University of Zaragoza, Pedro Cerbuna 12, 50009 Zaragoza, Spain; jorgepb@unizar.es (J.P.-B.); becalvo@unizar.es (B.C.); nmedrano@unizar.es (N.M.); pemar2@unizar.es (P.A.M.)

**Keywords:** impedance spectroscopy, phase sensitive detection, programmable quadrature oscillator, CMOS integrated circuits, low-power

## Abstract

This paper presents a low-power fully integrated quadrature signal generator for system-on-chip (SoC) impedance spectroscopy applications. It has been designed in a 0.18 μm-1.8 V CMOS technology as a self-contained oscillator, without the need for an external reference clock. The frequency can be digitally tuned from 10 to 345 kHz with 12-bit accuracy and a relative mean error below 1.7%, thus supporting a wide range of impedance sensing applications. The proposal is experimentally validated in two impedance spectrometry examples, achieving good magnitude and phase recovery results compared to the results obtained using a commercial LCR-meter. Besides the wide frequency tuning range, the proposed programmable oscillator features a total power consumption lower than 0.77 mW and an active area of 0.129 mm^2^, thus constituting a highly suitable choice as stimulation module for instrument-on-a-chip devices.

## 1. Introduction

Recent advances in microsensing techniques are leading to a growing need for on-chip electronic instrumentation, not only providing the required performances but also simultaneously complying with the constraints of low power and compact size, to fully satisfy the emerging market demands and potential applications of portable and wearable sensing devices. New transduction techniques in micro-integrated sensors include resonant detection and complex impedance characterization, as in surface acoustic wave sensors [1], gas sensors [2,3,4,5], laser interferometry [6], brain monitoring [7], non-invasive light detection [8] or biological impedance measurement [9,10]. For these transducers, a suitable interrogation approach, which presents advantages compared to other electronic readout techniques due to its characteristics, is synchronous demodulation.

**Figure 1:**
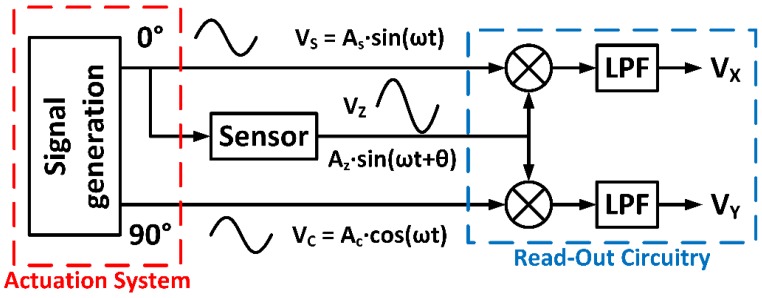
Conceptual scheme of the phase sensitive detection (PSD) technique.

Synchronous demodulation [11,12] can be implemented in CMOS technology [13,14] using phase sensitive detection (PSD) or quadrature modulators, which extract the signal amplitude and phase information at a specific frequency $f_{o}$ while noise signals at other frequencies are rejected. Figure 1 illustrates the conceptual scheme of a dual PSD module. A device sensor is excited by an input signal $V_{S}=A_{S}\sin\left(\omega t\right)$, providing an output $V_{Z}=A_{Z}\sin\left(\omega t+\theta \right)$ that is next multiplied by two 90 degrees shifted reference signals also with frequency $f_{o}$. The resulting mixer outputs are finally low-pass filtered, obtaining two DC values $V_{X}$ and $V_{Y}$, proportional to the processed signal:

(1) $$V_{X}\propto f\left(A_{Z},\theta \right)\;V_{Y}\propto f\prime \left(A_{Z},\theta \right)$$

so that the magnitude and phase, or equivalently, the real and imaginary part of the sensor impedance can be obtained.

Accordingly, in dual PSD-based conditioning electronics, the stimulation system requires the generation of two 90 degrees shifted signals $V_{S}$ and $V_{C}$, so that one of them is also used as the sensor excitation. Most of microelectronic implementations of synchronous demodulators integrate the read-out circuit together with the transducer in the same chip, but leave the stimulation system out of the integrated circuit, using external signal generators, thus increasing size, power consumption and complexity.

Therefore, a fully integrated PSD instrument will require the design of a self-contained suitable integrated stimulation system. This paper presents the implementation and experimental results of a versatile CMOS signal generation circuit suitable for portable PSD applications. Preliminary simulation results have been previously presented in [15]. To be self-contained, it is based on a digitally programmable analog quadrature sinusoidal oscillator. It generates two sinusoidal signals in quadrature, whose frequency is digitally controlled by a 12-bit custom digital-to-analog (DAC) architecture over a wide range up to hundreds of kHz, covering most typical impedance sensor operation. It has been fabricated in a 0.18 µm-1.8 V CMOS technology, featuring low-power and compact size, to be suitable for applications in portable on-chip systems, and it has been experimentally characterized as stimulation block in two impedance spectroscopy (IS) applications, from structural characterization to bio-impedance measurement.

The paper is organized as follows. Section 2 describes the proposed stimulation system, explaining its basic blocks and its implementation. Section 3 summarizes the experimental results of the fabricated prototype. Section 4 presents the experimental results for two IS applications. Finally, conclusions are drawn in Section 5.

## 2. Quadrature Oscillator

**Figure 2:**
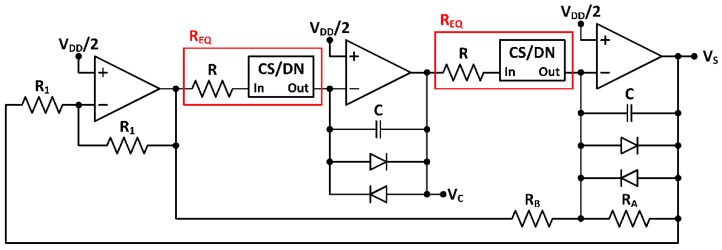
Proposed programmable quadrature oscillator topology.

The proposed quadrature sinusoidal oscillator is based on an analog core implementation. It provides low-voltage low-power compatibility with a CMOS hardware efficient architecture. To achieve a precise trimming over a wide frequency range, this architecture incorporates custom digitally programmable blocks, so that can be easily adjusted according to the target application. The oscillator topology is shown in Figure 2. It is based on a single supply active-RC two integrator loop using three operational amplifiers (OpAmps) as active cells, two capacitors and six resistances: resistors $R_{A}$, $R_{B}$ and $R_{1}$ are passive, while $R_{EQ}$ are identical active resistors, respectively conformed by a passive resistor R in series with a digital control cell based on a current summing/division network (CS/DNs) [15].

This oscillator generates a quadrature signal pair ($V_{S}$, $V_{C}$) with a phase shift of 90° [15]. By direct analysis of the first integrator loop, the relation between signals $V_{S}$ and $V_{C}$ is

(2) $$V_{S}=sCR_{EQ}V_{C}$$

Analogously, analyzing the second integrator loop,

(3) $$V_{C}=V_{S}\frac{R_{EQ}}{R_{A}R_{B}}\left(R_{A}-R_{B}-sCR_{A}R_{B}\right)$$

Hence, from (2) and (3), the characteristic equation of the system is

(4) $${\left(\omega CR_{EQ}\right)}^{2}-j\omega CR_{EQ}{}^{2}\frac{\left(R_{A}-R_{B}\right)}{R_{A}R_{B}}-1=0$$

Thus, the oscillation condition is given by

(5) $$-j\omega CR_{EQ}{}^{2}\frac{\left(R_{A}-R_{B}\right)}{R_{A}R_{B}}=0\Rightarrow R_{A}=R_{B}$$

and the oscillation frequency $f_{o}$ is

(6) $${\left(\omega CR_{EQ}\right)}^{2}=1\Rightarrow f_{o}=\frac{\omega_{o}}{2\pi }=\frac{1}{2\pi CR_{EQ}}$$

so that both characteristics are independently controllable. In practice, $R_{A}$ must be slightly higher than $R_{B}$ to guarantee oscillation. Combining (2) and (6), it is straightforward that resistors $R_{EQ}$ must vary simultaneously to keep constant the output amplitude ratio for the two output quadrature signals

(7) $$\left|\frac{A_{V_{C}}}{A_{V_{S}}}\right|=\frac{1}{\omega_{o}CR_{EQ}}=1$$

### 2.1. Active Cells Design

#### 2.1.1. Operational Amplifier

**Figure 3:**
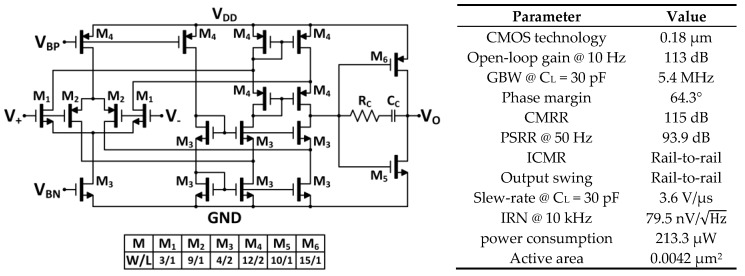
Operational amplifier schematic topology with transistor sizes in µm, and main post-layout simulated performances.

A two-stage OpAmp (Figure 3) has been specifically designed to accomplish high gain, high unity gain frequency, and class AB output to enhance the driving capability with optimum power consumption. The input stage is a folded cascode operational transconductance amplifier (OTA), which develops high gain with a simple single-stage topology, thus being suitable for low-voltage operation. The output is a class AB push-pull buffer. This additional stage provides rail-to-rail operation, while adds extra gain to the OpAmp. Stability is guaranteed through classical $R_{C}-C_{C}$ Miller compensation. The main post-layout simulated performances for a 1.8 V-0.18 µm CMOS implementation are summarized in Figure 3.

#### 2.1.2. Current Summing/Division Network

**Figure 4:**
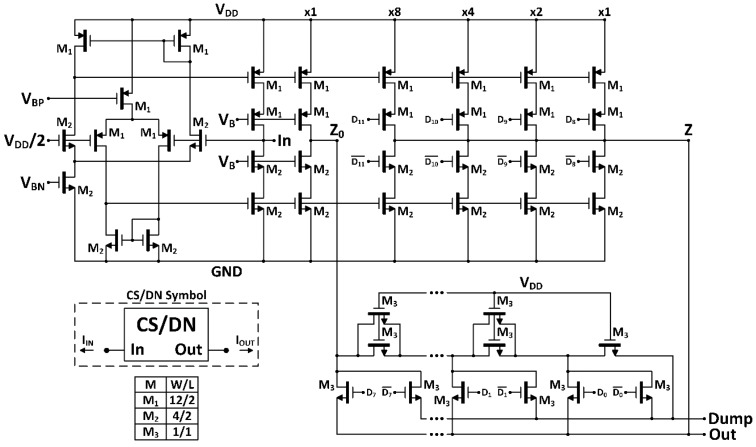
CS/DN schematic topology and transistor sizes in µm.

The digitally programmable block is based on a current summing/division network (CS/DN) approach [15], that digitally controls the current to be delivered to the virtual ground node of single-OpAmp trans-impedance amplifiers, setting a linear programmable gain in the case of resistive feedback, or a linear programmable time constant in the case of capacitive feedback. The CS/DN scheme is shown in Figure 4.

A n=12 bit resolution has been chosen to attain a good tradeoff between tuning range, resolution, power and area consumption. The CSN provides the m=4 most significant bits (MSB). It is basically a wide-swing class AB second generation current conveyor (CCII^+^) topology, where terminal *X* is the input CS/DN current terminal. The input common mode voltage $V_{X}=V_{Y}$ is fixed by setting terminal *Y* to $V_{DD}/2$.

**Figure 5:**
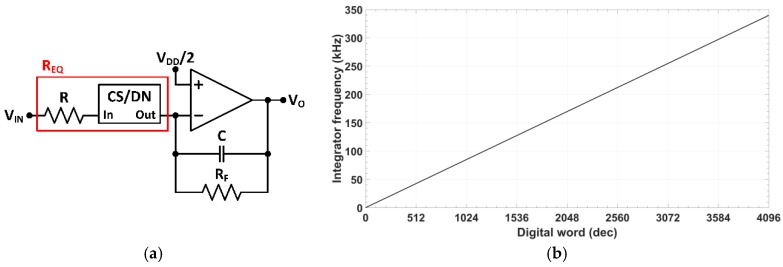
(**a**) Active-RC integrator built around the proposed OpAmp and CS/DN; and (**b**) simulated integrator characteristic frequency.

The *Z* output stage ($I_{Z}=I_{X}$) is replicated to achieve 16 identical unity gain currents (15 *Z* branches and *Z*_0_ branch, Figure 5). So, *Z* is directly decoded to a binary representation by driving the gates of the corresponding cascode output transistors to attain currents ×1, ×2, ×4, ×8 with bits $D_{8}$ to $D_{11}$, respectively. The *Z*_0_ unitary current is the input current of the l=8 least significant bit (LSB) segment: a MOS R/2-R ladder implemented with NMOS transistors of identical size. The last branch of the ladder is connected to the Dump node, and therefore there is no offset current for the zero digital input. Classical MOS ladders are the best solution in terms of size and power consumption for resolutions up to 8 bit, which can be increased up to 10 bit with careful layout techniques at the cost of area [16]. Hence, the resolution of each segment of the CS/DN was set to keep a good tradeoff between circuit performance, complexity and power consumption.

Therefore, the full-scale current is

(8) $$I_{FS}=2^{m}\cdot I_{in}=2^{4}\cdot I_{in}$$

and the LSB current is

(9) $$I_{LSB}=\frac{I_{FS}}{2^{n}}=\frac{I_{FS}}{2^{12}}=\frac{I_{in}}{2^{l}}=\frac{I_{in}}{256}$$

Thus, the total output analog current is given by

(10) $$I_{out}=I_{LSB}\overset{n-1}{\underset{j=0}{\sum }}D_{j}2^{j}=\frac{I_{in}}{256}\overset{11}{\underset{j=0}{\sum }}D_{j}2^{j}=\frac{I_{in}}{256}D\left(12\right)$$

where $D_{j}$ are the coefficients of the 12-bit digital control word D(12) representing the input binary code. Hence, a linear relationship is obtained between the digital programming word and the output current.

Note that the Dump node of the MOS ladder (Figure 4) must be connected to a virtual $V_{DD}/2$ ground to preserve the output terminals symmetry, ensuring a right current division.

This 12 bit CS/DN structure has been implemented in a 1.8 V-0.18 µm CMOS process, with the transistor sizes and parameter values indicated in Figure 4. It features as main post-layout performances: 12-bit resolution with linearity errors below ±0.5 LSB, static power consumption lower than 44 µW, an intrinsic bandwidth of 6 MHz and an active area of 0.0085 mm^2^.

The cell behavior has been tested in a first order analog integrator (Figure 5a), using the OpAmp shown in Figure 3 as active component. When this module is connected in series with a linear resistor R as shown in Figure 5a, the input current to the programmable module is $I_{in}=V_{in}/R$, generating a current at the output given by (7) which is transferred to the integrator feedback impedance ($C∥R_{F}$). The voltage transfer function for $R_{F}\gg R$ is

(11) $$\frac{v_{out}}{v_{in}}≅\frac{D\left(12\right)}{256sCR}=\frac{1}{sCR_{EQ}}$$

and the combined R-CS/DN module behaves as a programmable resistor, with an equivalent resistance

(12) $$R_{EQ}=\frac{256R}{D\left(12\right)}$$

Thus, the transfer function of an ideal integrator is recovered, but exhibiting a non-inverting behavior due to the 180° phase shift introduced in the current follower ($I_{X}$, $I_{Z}$) of the CCII^+^ in the CS/DN. The integrator characteristic frequency is given by

(13) $$f_{o}=f_{int}=\frac{D\left(12\right)}{256\cdot 2\pi CR}$$

showing a linear relationship with the 12-bit digital control word. Theoretical frequency range varies from $f_{o,min}=0$ to $f_{o,max}=4095/\left(256\cdot 2\pi CR\right)\approx 16/2\pi CR$ in steps of $f_{o,step}=1/\left(256\cdot 2\pi CR\right)$. Figure 5b shows the simulated integrator characteristic frequency over the whole digital range, setting R=250 kΩ, $R_{F}=1$ MΩ and C=30 pF, to attain a theoretical frequency range from 0 to 339.5 kHz, with a resolution of 82.9 Hz. Maximum relative error of the integrator characteristic frequency is below 0.7%.

Therefore, in active-RC systems, with characteristic frequencies given by ~1/RC, this cell brings out an accurate frequency trimming with linear dependence on the digital input code.

### 2.2. Quadrature Oscillator Design

To attain a fully integrated 1.8 V-0.18 µm CMOS stimulation system operating up to hundreds of kHz, the passive components are set to the values shown in Table 1. Resistors are implemented with a high resistive polysilicon (HRP) layer and capacitive elements are metal-insulator-metal (MIM) capacitors. Floating diodes in the integrator loops are standard cells provided by the CMOS technology, built in a P-well, each having W=L=10 µm size. They are used to avoid saturation of the output signal amplitudes [17].

**Figure 6:**
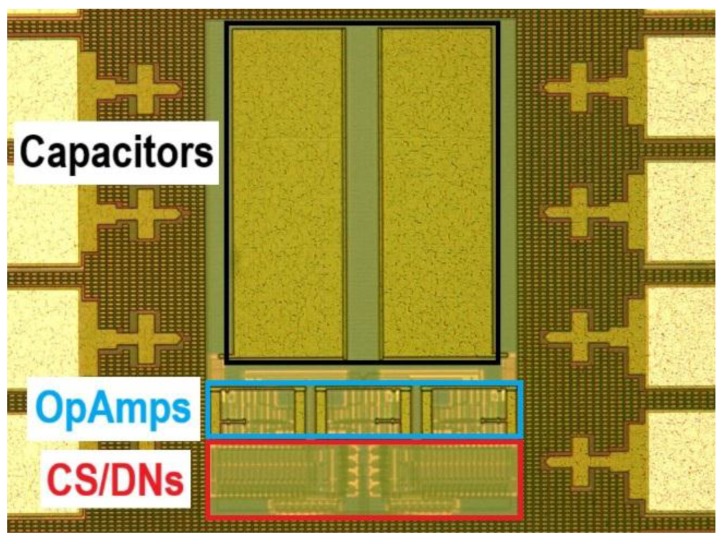
Quadrature oscillator prototype (active area: 283 µm × 455 µm).

Figure 6 shows a microphotograph of the fabricated prototype. Its silicon area is 0.129 mm^2^, of which about 70% corresponds to the capacitors.

## 3. Experimental Performances

### 3.1. Test Setup

**Figure 7:**
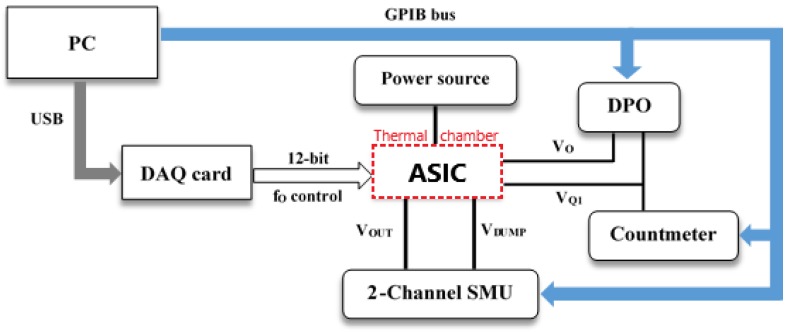
Experimental setup block diagram.

**Figure 8:**
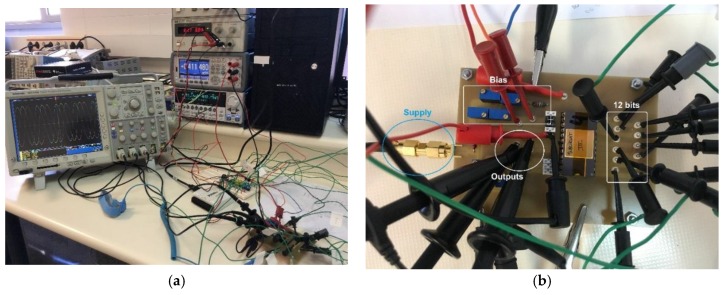
Experimental setup: (**a**) General view of the instrumentation used; and (**b**) A detail of the ASIC connected to the circuit board, indicating the supply and bias lines, the output signals, and the 12 bit digital input.

Figure 7 shows the block diagram of the experimental setup used to test the fabricated quadrature oscillator. A Keysight E3611A power supply (Keysight Technologies, Santa Rosa, CA, USA) provides the 1.8 V supply voltage required by the circuit. Frequency programming is provided by a NI USB-6009 Data Acquisition Card (National Instruments, Austin, TX, USA) that controls the 12-bit registers in the device. A Keithley 2602A two-channel SourceMeter Unit (Keithley Instruments Inc., Cleveland, OH, USA) sets both the Out and Dump node voltages to a virtual ground value (0.9 V), while the oscillator dynamic characteristics are registered through a Tektronix 4104 Digital Phospor Oscilloscope (Tektronix Inc., Cleveland, OH, USA). A Keysight 53132A Universal Counter (Keysight Technologies, Santa Rosa, CA, USA) accurately measures the oscillation frequency. Characterization system is controlled by a computer using Universal Serial Bus (USB) and General Purpose Interface Bus (GPIB) communication standards. Figure 8 shows some photographs of the experimental measurement setup.

### 3.2. Experimental Results

**Figure 9:**
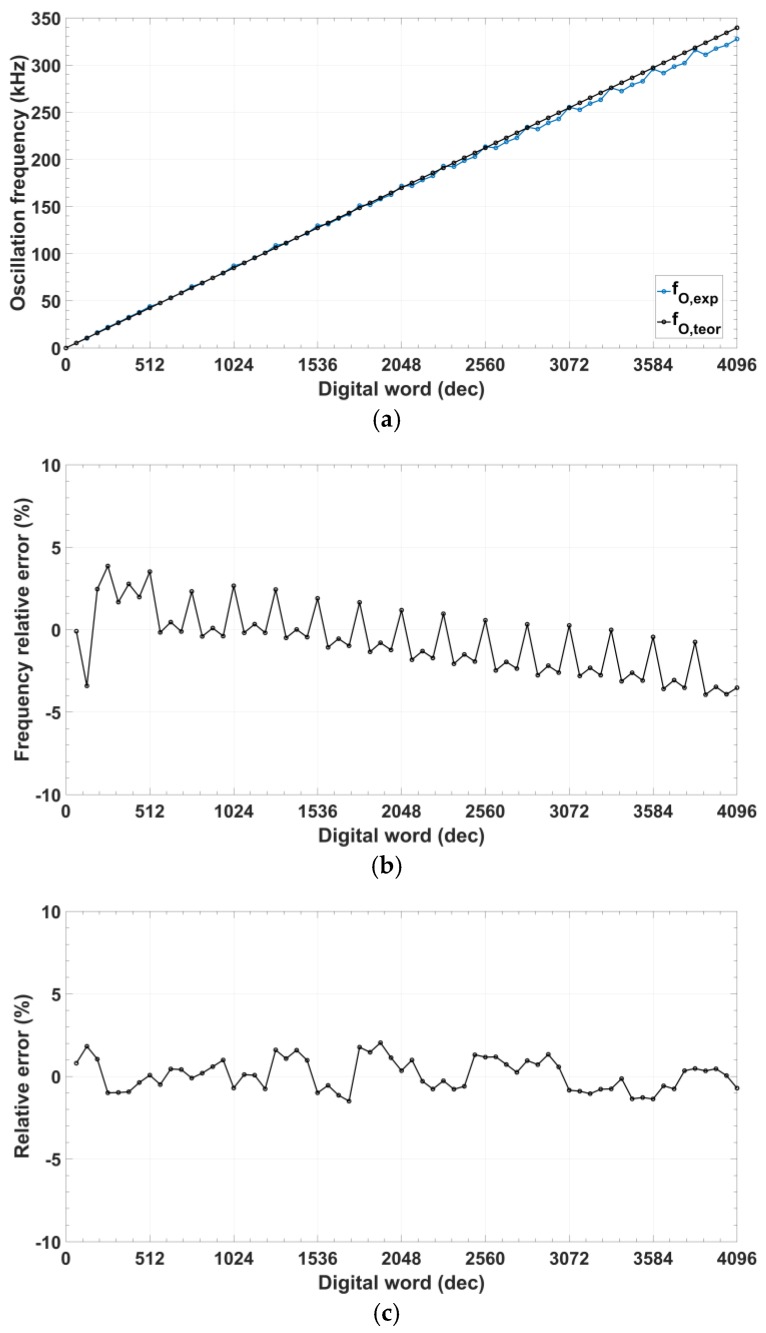
Oscillator dynamic performance measurements: (**a**) Oscillation frequency; (**b**) Frequency relative error; (**c**) Phase relative error.

Figure 9a shows the oscillation frequency measured as a function of the 12-bit digital control. Effective frequency ranges from 10 kHz for a digital value of #80 to 345 kHz for a register value of #FFF, with a maximum relative error below 4% and absolute mean error lower than 1.7% (Figure 9b). Frequency peak errors are due to activation/deactivation of the most significant bits (associated to the CCII^+^) in the programmable cell, resulting in changes in the input/output impedance of the CS/DN block. Figure 9c shows the phase error for the quadrature output signals, which remains lower than 2% with an absolute mean error below 0.84%.

**Figure 10:**
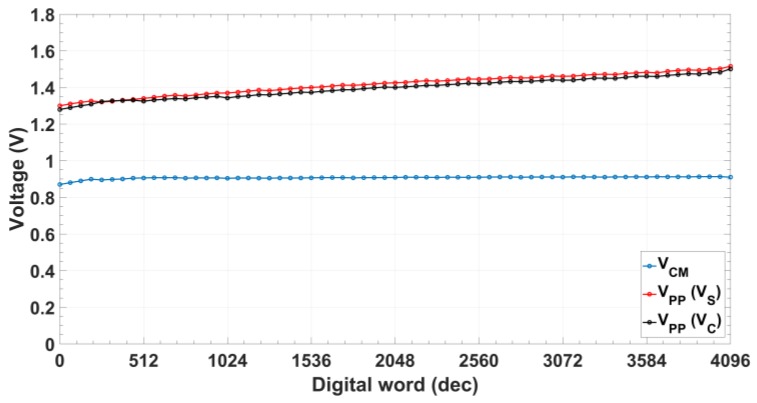
Oscillator common mode (V_CM_) and peak-to-peak (V_PP_) voltage of quadrature signals.

**Figure 11:**
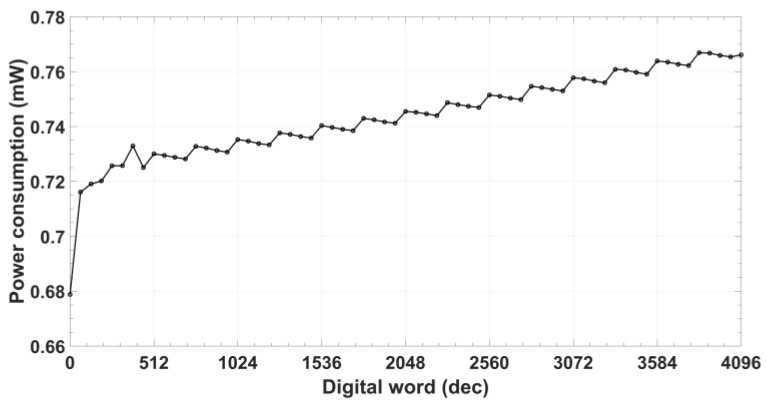
Oscillator power consumption.

Common mode voltage (Figure 10) remains constant to $V_{DD}/2$ (0.9 V) for the whole digital range with an error below 0.4%, while peak-to-peak voltage varies from 1.32 to 1.52 V with the digital control code for signal $V_{S}$ and from 1.28 to 1.50 V for signal $V_{C}$. Maximum difference between them is 0.027 V (~2% deviation). Due to the CS/DN architecture, the power consumption is code-dependent, being always lower than 770 µW (Figure 11).

**Figure 12:**
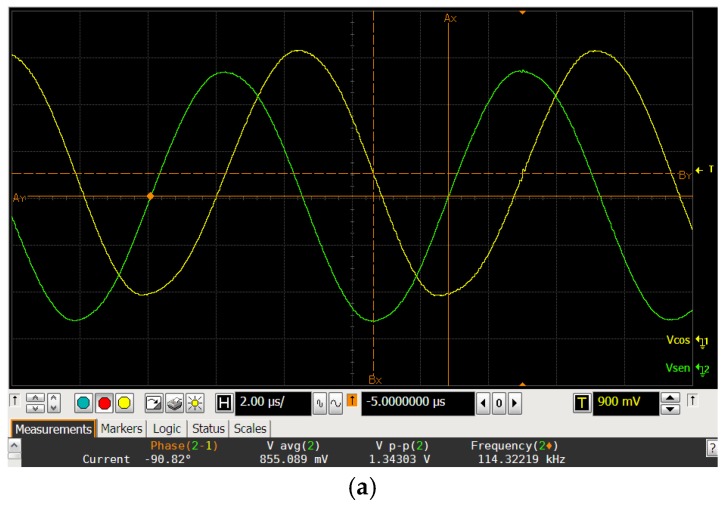

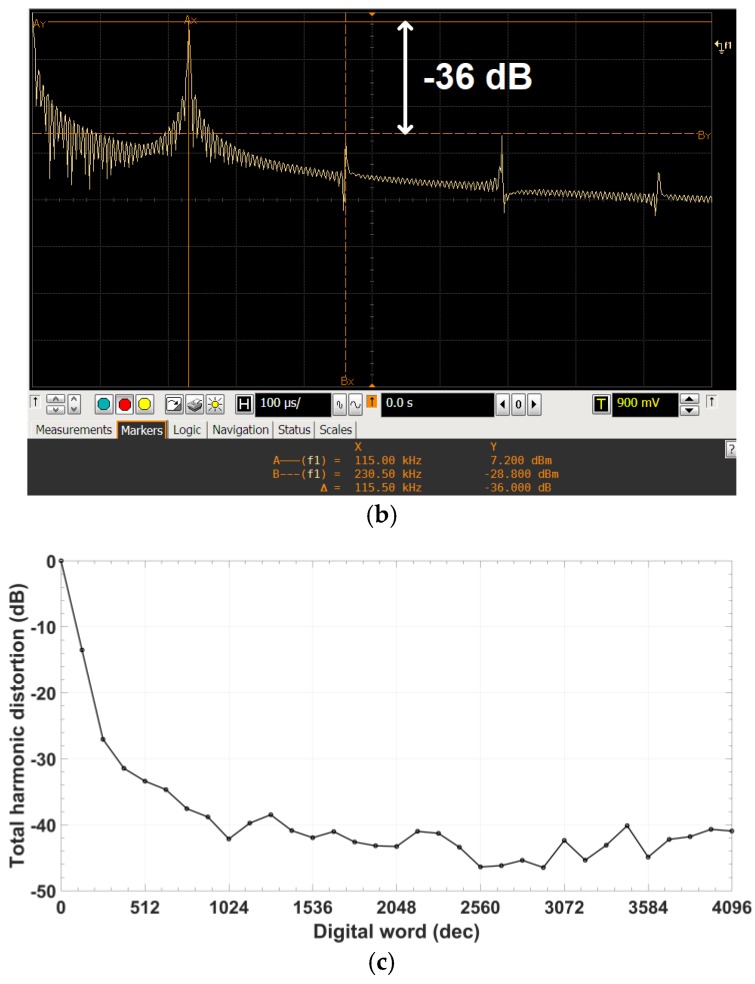
Output quadrature signals measurements: (**a**) Screenshot of the oscilloscope for the digital code #500; (**b**) Spectral analysis of the output signal for the digital code #500; and (**c**) Total harmonic distortion of the output quadrature signals.

Figure 12a shows an oscilloscope screenshot of the output quadrature signals for the digital word #500, and Figure 12b shows its spectral analysis, using a Hanning window. These signals oscillate at a frequency of 114.3 kHz, with a total harmonic distortion (THD) of −36 dB. Figure 12c shows the THD value along the digital range. Finally, Table 2 summarizes the main performances of the fabricated oscillator.

**Figure 13:**
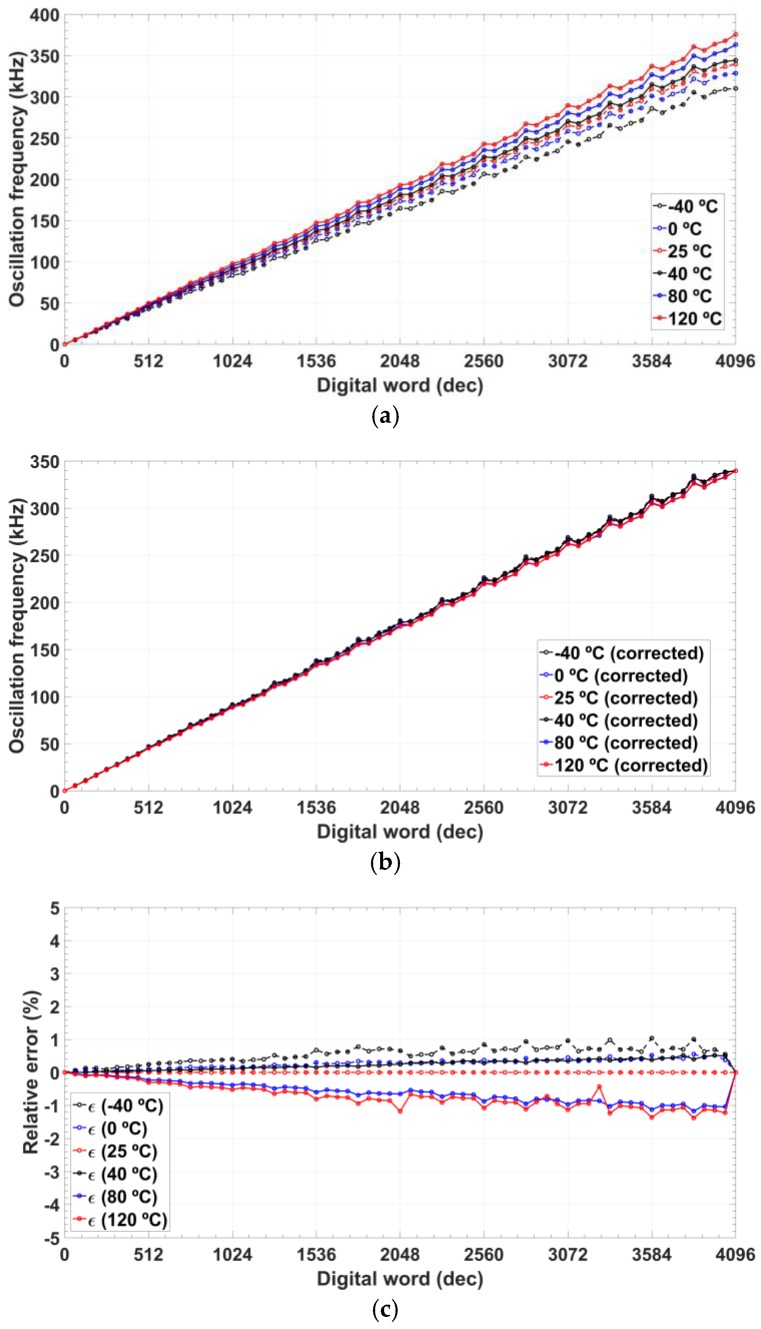
Oscillation frequency for a temperature range from −40 to 120 °C: (**a**) measured; (**b**) slope error corrected; and (**c**) relative error after correction.

The drift in the value of the programmed frequency due to the effects of the temperature in the oscillator has been characterized using a thermal chamber Fitoterm 22E from Aralab (Aralab Headquarters, Sintra, Portugal) in the range from −40 to 120 °C (Figure 13a). The proposed oscillator shows a slope error in the frequency programmability, lower than 12.9% from room temperature up to 120 °C and less than 8.7% for variations from room temperature down to −40 °C. Figure 13b shows the frequency programmability after the slope error has been corrected. Then, the maximum relative error is reduced down to 1.5% and absolute mean error to below 0.71% (Figure 13c).

Comparing these performances with other previous works in the literature [18,19,20], only [18] presents a monolithic CMOS digitally programmable quadrature oscillator, but its resolution is limited to 6-bit, and the oscillation frequency (0.3 to 1.3 MHz) is nonlinear and inversely proportional to the digital word. In [19], the programmability resolution is 12-bit and the control is linear for a frequency range from 48 to 92 kHz, but it only integrates the active cell while the programmable element is implemented using external components, being non-compatible with low-voltage low-power operation. In [20], the oscillation frequency (from 29 to 230 kHz) lies within our target frequency range, but with non-linear analog control: the oscillation frequency is set by changing a transconductance parameter $g_{m}$ from 14 to 2.8 mS, while relies on external integrating 1 nF capacitors not suitable for on-chip solutions. Thus, to the best of the authors’ knowledge, this is the first fully integrated high resolution quadrature sinusoidal oscillator on the proposed frequency range with linear digital control compatible with the two key requirements of portable systems: low-voltage low-power operation and reduced size.

## 4. Application to Impedance Characterization

**Figure 14:**
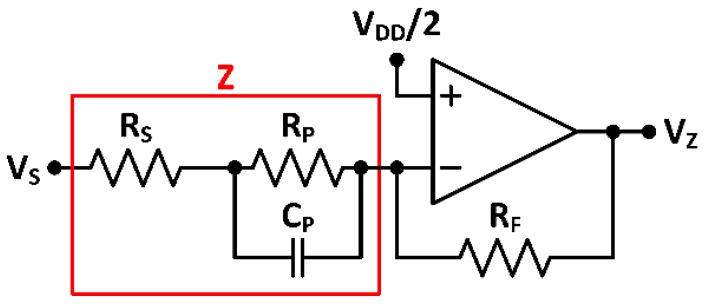
Test impedance with an auto-balancing bridge.

The suitability of the proposed quadrature oscillator as signal source for an IS micro-instrument is herein analyzed. For this, the selected test impedance configuration emulates the real impedance configuration known as Randles cell [10]: the target impedance Z is connected to a standard setup circuit consisting on an OpAmp, configured as an auto-balancing bridge with a feedback resistor $R_{F}=295\;k\Omega$ (Figure 14).

For this circuit, when a sinusoidal signal $V_{S}=A_{S}\sin\left(\omega t\right)$ excites the input of impedance Z, the output signal is given by:

(14) $$V_{Z}=-\frac{R_{F}}{Z}V_{S}=-\frac{R_{F}}{\left|Z\right|}A_{S}\sin\left(\omega t+\theta \right)$$

In this way, it is possible to recover both magnitude and phase (related to the resistance and reactance components of the complex impedance), by next applying a synchronous quadrature demodulator using the signals provided by the proposed microelectronic stimulation circuit (Figure 1).

**Figure 15:**
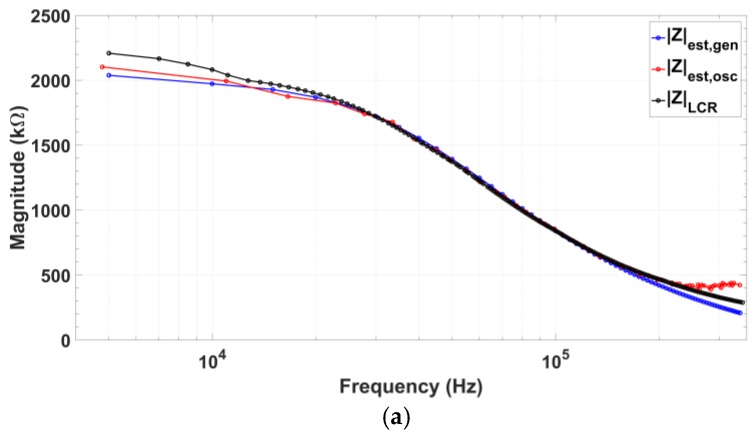

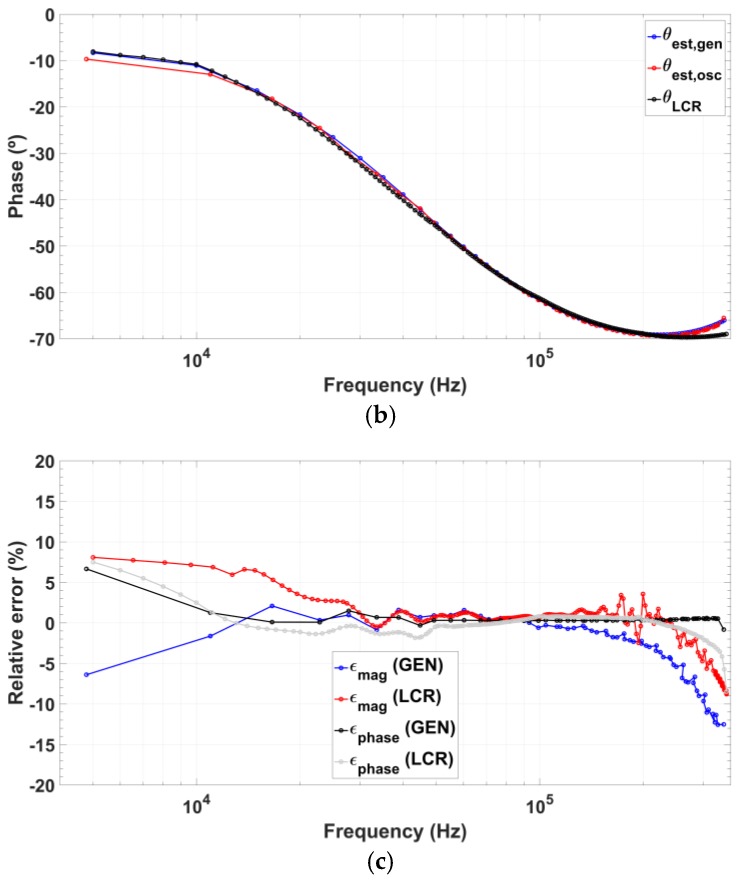
Impedance measurement applying input quadrature signals generated by the fabricated oscillator prototype (red), commercial waveform generator (blue) and commercial 8101G LCR meter (black): (**a**) Impedance magnitude recovery; (**b**) Impedance phase recovery; and (**c**) Recovery errors for both magnitude and phase using the proposed integrated oscillator compared to the results applying the commercial AWG and the values measured by the LCR-meter.

The first test impedance components selected are (Figure 14) $R_{S}=6.78\;k\Omega$, $R_{P}=1.974\;M\Omega$, and $C_{P}=2\;\mathrm{pF}$. Oscillator reliability was verified by comparing the obtained results to those achieved using a commercial 33522A AWG arbitrary waveform generator (Keysight Technologies, Santa Rosa, CA, USA) as signal source. Recovered impedance magnitude and phase are shown in Figure 15a,b. Besides, the feasibility of applying this technique for impedance characterization was verified using a GW-Instek 8101G LCR Meter (Good Will Instrument, Taipei, Taiwan). LCR measurement results in impedance magnitude and phase recovery are also included in Figure 15a,b. Magnitude errors are below 7% up to 330 kHz and phase errors are below 6% up to 340 kHz (Figure 15c). Absolute mean errors using the presented oscillator compared to the LCR-meter measurements are 2.21% for impedance magnitude and 1.17% for phase readout.

**Figure 16:**
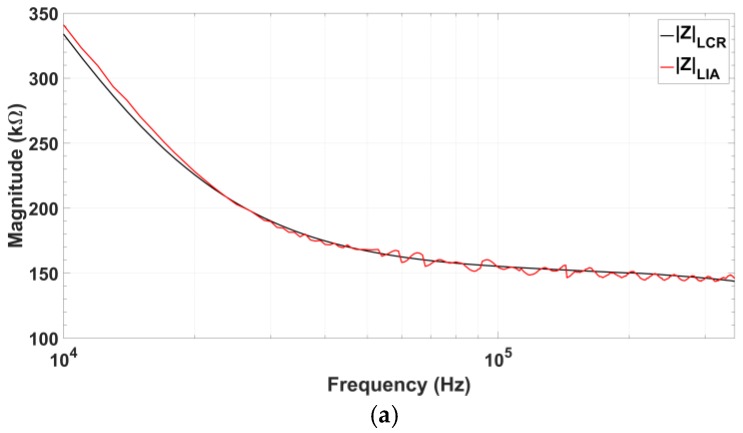

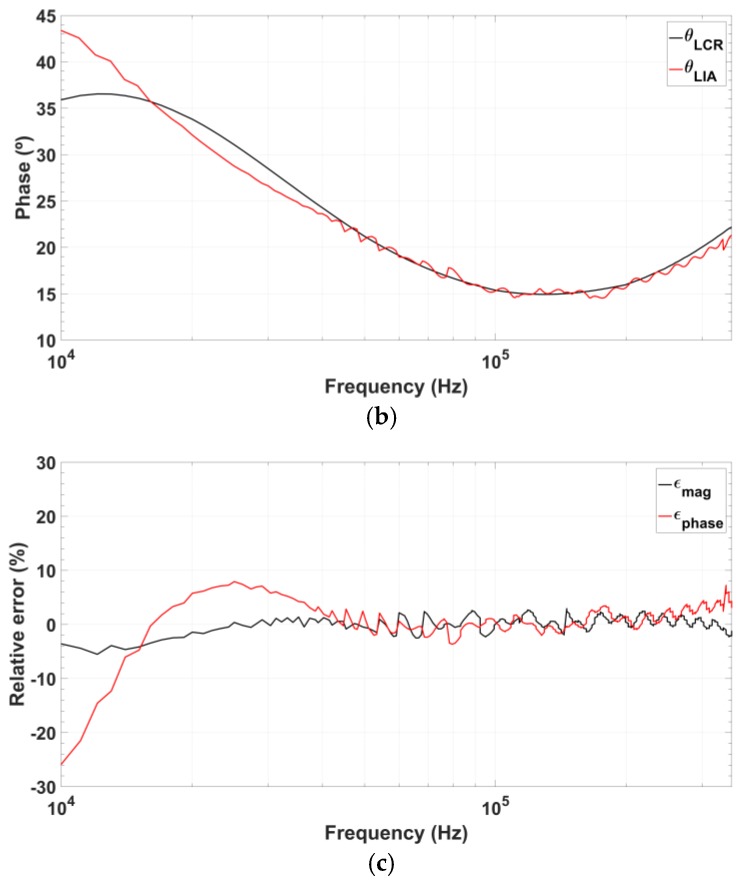
Comparison of the impedance values measured using the signals from the proposed quadrature oscillator prototype (red) and those obtained using the commercial 8101G LCR-meter (black) for impedance values measured in protein detection: (**a**) Recovered impedance magnitude; (**b**) Recovered impedance phase, and (**c**) Relative error of both magnitude and phase.

Next, the designed oscillator has been tested as a stimulation module in a biological impedance measurement system for protein detection. Complex impedance component values were selected according to real measurements shown in the literature [10]: $R_{S}=149.1\;k\Omega$, $R_{P}=431\;k\Omega$, $R_{F}=429\;k\Omega$, and $C_{P}=57\;\mathrm{pF}$. For a suitable comparison, oscillator amplitude has been limited to 100 mV, considered as a typical input signal amplitude for this application. Figure 16 shows the impedance magnitude and phase obtained using the integrated oscillator compared to the value obtained using the commercial LCR-meter. Absolute recovery error of impedance magnitude always remains below 5%, while phase recovery error is below 5% for a frequency range from 34 to 337 kHz (90% of the oscillator total frequency range).

**Figure 17:**
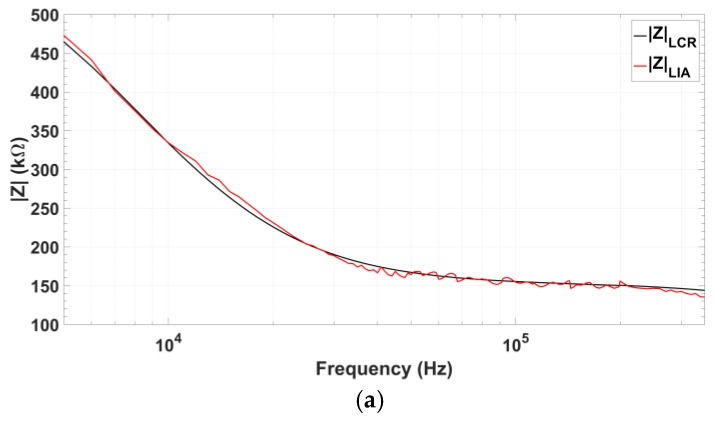

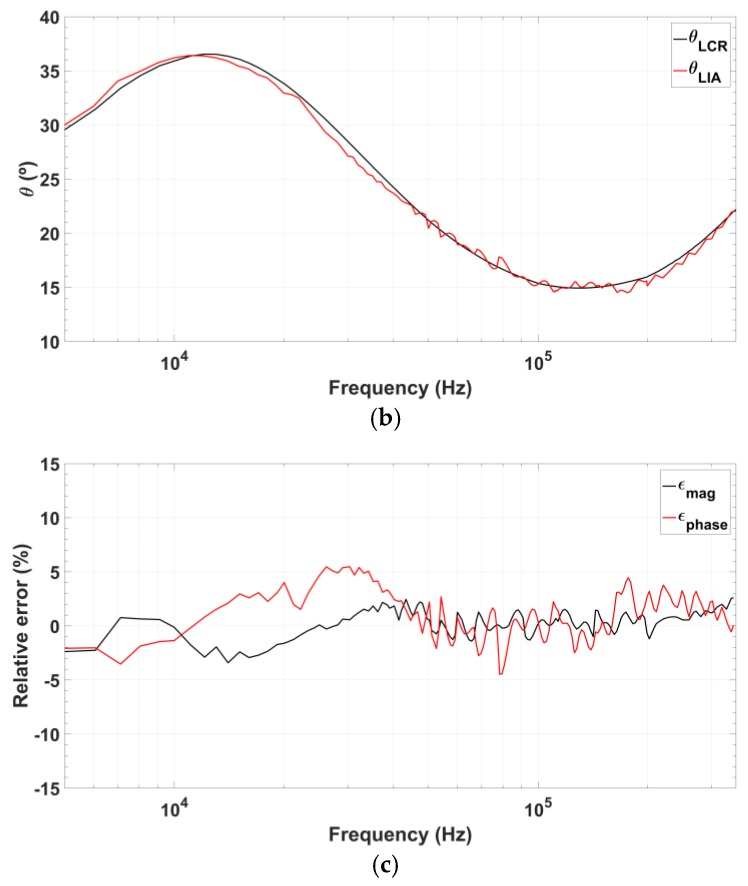
Comparison of the impedance values measured using the signals from the proposed quadrature oscillator prototype (red) and those obtained using the commercial 8101G LCR-meter (black) for impedance values measured in protein detection, but employing a different gain setup for the three frequency ranges: low (5–50 kHz), medium (50–250 kHz), and high (250–350 kHz). (**a**) Recovered impedance magnitude; (**b**) Recovered impedance phase, and (**c**) Relative error of both magnitude and phase.

These measurements have been done at fixed 100 mV_pp_ excitation and nominal fixed 2 gain for the Randles cell at medium frequency range, but applied over the entire frequency range. However, it has to be noted that at the low frequency range impedance increases, reducing the system gain and thus the output signal, modifying the measurement conditions. On the contrary, at the high frequency range, impedance under test decreases, and therefore system gain must be reduced to keep the voltage measurement conditions almost constant. For an in-depth study of those effects, we have made new measurements with different gain conditions associated to the three frequency (low, medium, high) ranges: a gain of 4.5 in a 10 to 50 kHz span, a gain of 2 in a 50 to 250 kHz span, and a gain of 1.5 from 250 to 350 kHz. Figure 17 shows these measurements, displaying errors below 5%. In this way, it is possible to perform a fast impedance coarse characterization at constant gain for the Randles cell, or an accurate characterization using a configurable gain cell.

## 5. Conclusions

A novel CMOS 1.8 V–0.18 µm digitally programmable analog quadrature oscillator has been designed to be used as a stimulation system for phase sensitive detection signal recovery applications. It has been fabricated and tested, showing a linear frequency control ranging from 10 to 345 kHz, total power consumption lower than 0.77 mW, and active area of 0.129 mm^2^. Experimental tests show good performance as a self-contained signal generator in general purpose impedance PSD-based measurement applications, as proved by the comparison with the results achieved using a commercial LCR-meter. By properly limiting the maximum signal amplitude, its suitability as actuator system in biological impedance characterization has been also tested using an impedance protein model, successfully recovering both module and phase. Global characteristics make this design a highly suitable choice as a signal generation module for instrument-on-a-chip devices. To the best of the authors’ knowledge, this is the unique self-contained quadrature stimulation system, low-voltage low-power compatible and featuring such high frequency resolution over such a wide linear tuning range. In addition, to further increase the frequency into the MHz range, the gain-bandwidth product and bandwidth of the active elements (OpAmps and CS/CD network) should be increased at the cost of jeopardizing power consumption, besides redesigning the nominal values of resistors and capacitors.

## Figures and Tables

**Table 1:** Oscillator design parameters.

Parameter	Value
*C*	30 pF
*R*_1_	50 kΩ
*R_A_*	80 kΩ
*R_B_*	75 kΩ
*R*	250 kΩ

**Table 2:** Oscillator measured performances.

Parameter	Value
Frequency span	330 kHz
Resolution (Step)	12 bit (~84 Hz)
Total harmonic distortion	<−36 dB
Peak-to-peak voltage	1.32–1.52 V
Power consumption	<0.77 mW
Active area	0.129 mm^2^
